# Editorial: Breast milk composition and infant metabolism

**DOI:** 10.3389/fnut.2023.1191358

**Published:** 2023-04-05

**Authors:** Botian Chen, Defu Ma

**Affiliations:** School of Public Health, Peking University Health Science Center, Beijing, China

**Keywords:** breast milk composition, infant metabolism, human milk, breastfeeding, formula milk

Nutrition in early life is of fundamental importance in an infant's future health. Breast milk is the first natural functional food for infants and the “gold standard” for infant feeding (1). Breast milk not only contains various nutrients suitable for infants' digestion and absorption, but also contains a variety of bioactive substances, such as immunoglobulins, hormones, oligosaccharides, and bacterial constituents (2). These functional components in breastmilk are known to exert a series of beneficial effects, including reduced risk from infections and promoting various aspects of postnatal development. Although the mechanisms underlying some of these benefits have been elucidated, the origins of others that have been reported, such as influence on neurological, immunological, and metabolic outcomes remain more obscure.

Breastmilk does not stand alone. Breast milk is produced by the mammary glands of future mothers and lactating mothers. The effect of the mother's diet, nutrition and health status, and environmental exposures on the composition of breast milk, and infant developmental outcome, are also issues that need to be further understood. Additionally, as a dynamic functional food for infants, human milk might adapt to meet infants' needs under preterm, infectious, and others.

In this Research Topic, we would like to present reviews and original articles covering the latest developments in studying breast milk composition, formula milk, and infant metabolism. Studies discussing the health issues mentioned above are suitable for consideration in this Research Topic. We hope that our Research Topic will contribute to deepening our knowledge in this area and provide the basis for creating new prophylactic and therapeutic standards.

In this special e-collection there are 13 papers covering the above mentioned aspects.

Breast milk contains a variety of bioactive components, and the composition of breast milk has always been the focus of attention in maternal and infant research. Ten papers out of 13 (76.9%) were related to this topic. Poulsen et al. performed metabolomic analysis of mature breast milk from mothers who delivered at term after 1 month and found that the breast milk metabolome was dynamic during maturation, which compensated for the deficiencies of mature milk studies. Lactopontin is a protein present in breast milk and related to neonatal immunity. The study by Zhu et al. described in detail the dynamic changes of Lactopontin in breast milk of newborn mothers at different gestational ages. Vitamin E is another component of breast milk that is related to infant immunity, but the content of vitamin E in breast milk during different lactation periods has not been systematically described. To clarify this issue, Xi et al. in their meta-analysis summarized the currently published articles in support of maternal and infant protective strategies regarding vitamin E. Breast milk sialic acid is very important for the development of the nervous system of infants. Previous studies generally believed that diet may have an impact on the content of sialic acid in breast milk. Xie, Xu et al. found no correlation between the content of sialic acid in breast milk and diet in their study of 33 mothers in Xiamen, China. Iodine is very important in neonatal neurodevelopment, and breast milk is the main way for newborns to obtain iodine. Guo et al. comprehensively evaluated the characteristics and predictors of breast milk iodine concentration (BMIC) and proposed the best predictor of BMIC. Breast milk exosomes have always been a hot topic in the study of breast milk composition. Cho et al. conducted a comprehensive analysis of breast milk exosomal miRNAs from obese mothers and pointed out the differences between them and those from normal weight mothers, which pointed out a new direction for the study of the effect of maternal obesity on infants.

The impact of the large number of bioactive substances in breast milk on the infant, including body composition, hormonal and cytokine profiles, and microorganisms and metabolites in feces, has been the focus of attention. Previous studies have shown that human milk oligosaccharides play an important role in the formation of intestinal microecology in infants, but few studies have identified the specific oligosaccharides. Data from the study of the Cambridge Infant cohort by Chichlowski et al. showed that higher concentrations of 2'fl and LNFP1 could promote the proliferation of bifidobacteria and could increase the abundance of intestinal flora in infants. Preterm birth is the leading cause of death in children under 5 years of age (3). Studies on the association between various breast milk components and the growth and development of preterm infants have always been a key part of maternal and infant research. Glucocorticoids play an important role in the regulation of hypothalamic-pituitary-adrenal (HPA) axis in infants, especially in premature infants. Previous studies have only focused on the role of breast milk Glucocorticoids (GCs) in very early preterm infants. The study by Muelbert et al. focused on the role of breast milk GCs in middle and late preterm infants, which was a supplement to the effect of breast milk GCs on preterm infants.

Whether there are cross-generational effects of lactation diet has been inconclusive. Vargas et al.'s study on mice showed that protein restriction during lactation led to cross-generation effects of metabolic dysfunction in mice, which provided new research ideas for subsequent studies. Bronchopulmonary dysplasia (BPD) is a serious chronic lung disease that affects the long-term health of newborns. The main pathogenic mechanism is oxidative stress (OS). The review by Yang et al. summarizes the variety of antioxidant factors contained in breast milk and suggests a new direction for the treatment of BPD.

Due to various reasons, many newborns partially or completely rely on various formulas (4). How to make formula more similar to breast milk has been one of the research hotspots in this field. At present, the underlying mechanisms of osteopontin (OPN), 2′-fucosyllactose (2′-FL) and docosahexaenoic acid (DHA) on neural cell development are still unknown. Xie, Zhang et al. showed that OPN, 2′-FL and DHA have the effect of promoting myelination of nerve cells, which can provide a theoretical basis for further optimization of infant formula. Fatty acid (FA), another major component of breast milk, may be affected by a variety of factors. Ni et al.'s study found that a variety of socio-demographic factors (e.g., maternal age, gestational weight, etc.) were related to total FA, while sn-2 FA content was almost not affected, which may provide ideas for the development of infant formula.

In summary, the results of the above studies and reviews represent a large amount of relevant data on the dynamic changes of human milk (HM) composition and its metabolism in infants. Despite all the available literature and evidence relating to this extremely important topic, the papers published in this e-book clearly demonstrate that there are still many aspects that need to be clarified and understood in relation to HM components and infant metabolism. After reading this book, some topics, such as the dynamics of HM composition, the effects of HM composition on infant metabolism, short-and-long term effects of breast milk composition on infant diseases, formula development based on breast milk composition, will become clearer to the reader and reinforce the belief that HM represents the best food for feeding all newborns, including preterm infants.

## Author contributions

DM wrote the introduction and the conclusion. BC wrote the central part with comments to the cited papers and references. All authors contributed to the article and approved the submitted version.
